# Intraluminal tracheal lipoma as a rare cause of dyspnoea in a dog

**DOI:** 10.1186/s13028-018-0413-5

**Published:** 2018-09-27

**Authors:** Kirsi Johanna Piirainen, Sanna Johanna Viitanen, Anu Katriina Lappalainen, Sari Helena Mölsä

**Affiliations:** Department of Equine and Small Animal Medicine, PL 57, Faculty of Veterinary Medicine, University of Helsinki, P.O. Box 57, 00014 Helsinki, Finland

**Keywords:** Airway, Canine, Lipoma, Neoplasia, Trachea

## Abstract

**Background:**

Tracheal tumors are rarely diagnosed in veterinary medicine and the majority of tracheal neoplasia reported in adult dogs are malignant. Intratracheal lipoma has not been previously reported in the veterinary literature.

**Case presentation:**

A 7-year-old Briard dog was evaluated for inspiratory dyspnoea and an inspiratory wheeze. Cervical radiographs and tracheoscopic examination revealed an intratracheal mass that was surgically removed. The dog has been asymptomatic after the surgery.

**Conclusions:**

Based on histopathology, the mass was diagnosed as lipoma. To the authors‘ knowledge, this is the first published report of an intratracheal lipoma in the veterinary literature.

## Background

Tracheal neoplasia is infrequently encountered in domestic animals and humans; the majority of which are malignant [1, 2]. The incidence of tracheal tumours in dogs and cats is not known but in human medicine the reported annual incidence ranges from 0.142 to 0.27 per 100,000 people [3, 4].

Tracheal tumours reported in dogs and cats include adenocarcinoma, carcinoma, extramedullary plasmacytoma, leiomyoma, fibrosarcoma, mast cell tumour, rhabdomyosarcoma and squamous cell carcinoma [5–10]. In addition to these, which are usually found in middle-aged or older animals, benign osteochondroma and osteochondromal dysplasia have been reported in dogs less than 2 years of age [11, 12]. Non-neoplastic lesions causing obstruction of the tracheal lumen including polyps, eosinophilic granulomas, zygomycosis and haematomas are also known to occur [13–16]. In humans, tracheal lipomas have also been reported rarely [17].

Dogs with an obstructing tracheal tumour typically present with clinical signs of inspiratory dyspnoea, panting, cough, auscultatory wheeze or collapse [1]. A tracheal mass can often be demonstrated in radiographs as the air in the tracheal lumen provides good contrast [1]. Computed tomography (CT) and tracheoscopy provide further information on the size of the tumour [1, 18]. CT may also further aid the evaluation of local and distant metastases and depth of invasion [18, 19]. Tracheoscopy is useful in evaluating the degree of tracheal lumen obstruction and mass appearance and allows fine-needle aspiration (FNA) or biopsy of the mass to obtain a cytological or histological diagnosis. Reported treatment options for tracheal tumours in dogs and cats include surgical tracheal resection, endoscopic snaring and radiation therapy [1].

Intraluminal tracheal lipoma has not been previously described in the veterinary literature. This case describes the successful surgical management of an endotracheal lipoma causing upper airway obstruction in a dog.

## Case presentation

A 7-year-old, 32 kg, neutered female Briard dog presented with inspiratory dyspnoea and an audible inspiratory wheeze particularly during exercise and after eating. The symptoms had been gradually worsening for a period of 3 months. There had been no cough or nasal discharge.

Mild inspiratory dyspnoea and increased inspiratory sounds during tracheal and laryngeal auscultation were noted at rest. Symptoms progressed to moderate to severe inspiratory dyspnoea under stress and an audible inspiratory wheeze became evident. Serum biochemistry revealed raised alkaline phosphatase activity (144 μ/L; reference interval < 95); other values were within normal range.

**Fig. 1:**
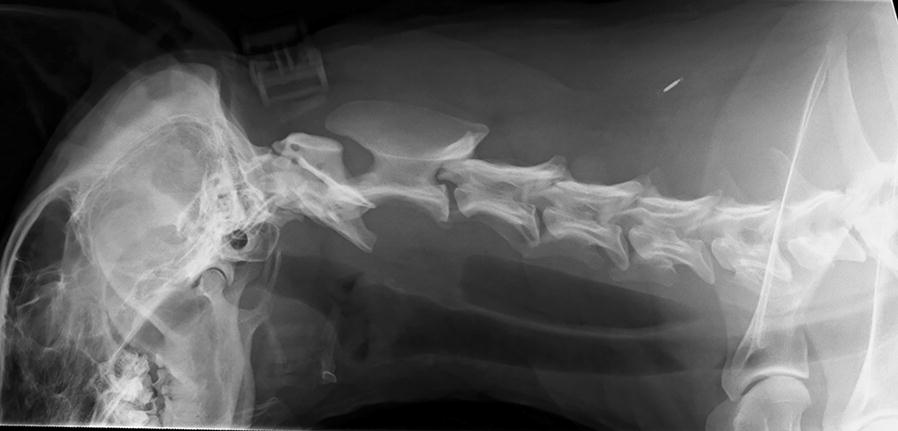
Left lateral cervical radiograph revealed a crescent shaped soft tissue opaque mass of 14 mm × 32 mm in the tracheal lumen at the level of the 5th and 6th vertebrae. The mass was at its widest in dorsal aspect of the trachea and extended to the opposite wall of the trachea

Left lateral cervical projection radiograph and radiographs of the thorax (right and left lateral and dorsoventral projections) were obtained. The dorsoventral projection included the caudal cervical trachea. In the left lateral cervical radiograph, a soft tissue opaque crescent shaped mass with long side dorsally was identified in the tracheal lumen at the level of the 5th and 6th cervical vertebrae (Fig. 1). The mass measured 14 mm × 32 mm, and the tracheal diameter was reduced at the site. The mass was not visible in the dorsoventral projection. The cervical and thoracic parts of oesophagus were markedly dilated and air-filled, most likely due to dyspnea. Otherwise the thoracic radiographs were unremarkable.

**Fig. 2:**
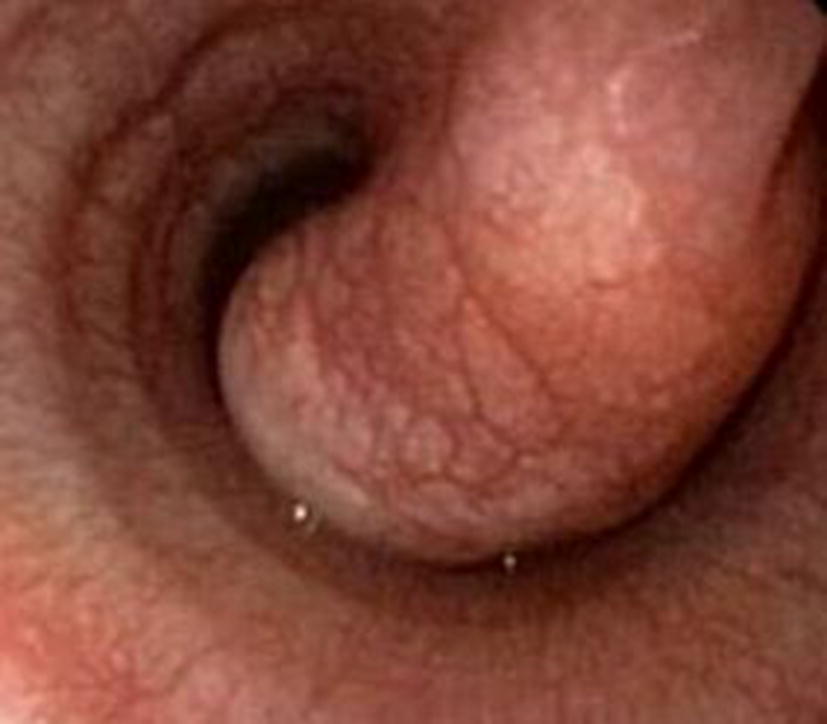
Endoscopic view of the mass in the tracheal lumen. The surface of the mass was smooth and its colour was the same as that of the tracheal epithelium

Tracheoscopy and bronchoscopy were performed under light anaesthesia with butorphanol (Torpudor, Richter Pharma AG) and propofol (PropoVet Multidose, Fresenius Kabi AB) with a 4.9-mm flexible endoscope (Olympus GIF-N180). An approximately 3 cm long intraluminal mass originating from the dorsal membrane of the trachea was detected approximately 11–12 cm from the larynx (Fig. 2). The base of the mass seemed to extend slightly to the left side of the dorsal membrane. FNA of the mass was performed using an endoscopic needle (Olympus NM-401L-0423, size 23G, diameter 0.6 mm and length 4 mm). Cytology of the samples revealed a scarce population of benign monomorphic mesenchymal cells. No signs of inflammation or malignancy were noted.

The surgery was performed 9 days later. The dog was pre-medicated with morphine (Morphin; Takeda Austria GmbH) and acepromazine (Plegicil; Pharmaxim) and anaesthesia was induced with propofol (PropoVet Multidose, Fresenius Kabi AB), ketamine (Ketaminol; Intervet) and midazolam (Midazolam Accord; Accord Healthcare Limited). Anaesthesia was maintained with a constant-rate infusion of fentanyl (Fentanyl-Hameln; Hameln Pharma Plus Gmbh). Intubation was performed under endoscopic guidance and an endotracheal tube was placed such that its cuff was distal to the mass. Cefazoline (Kefzol; Eurocept) was given intravenously 30 min before incision and every 90 min thereafter.

**Fig. 3:**
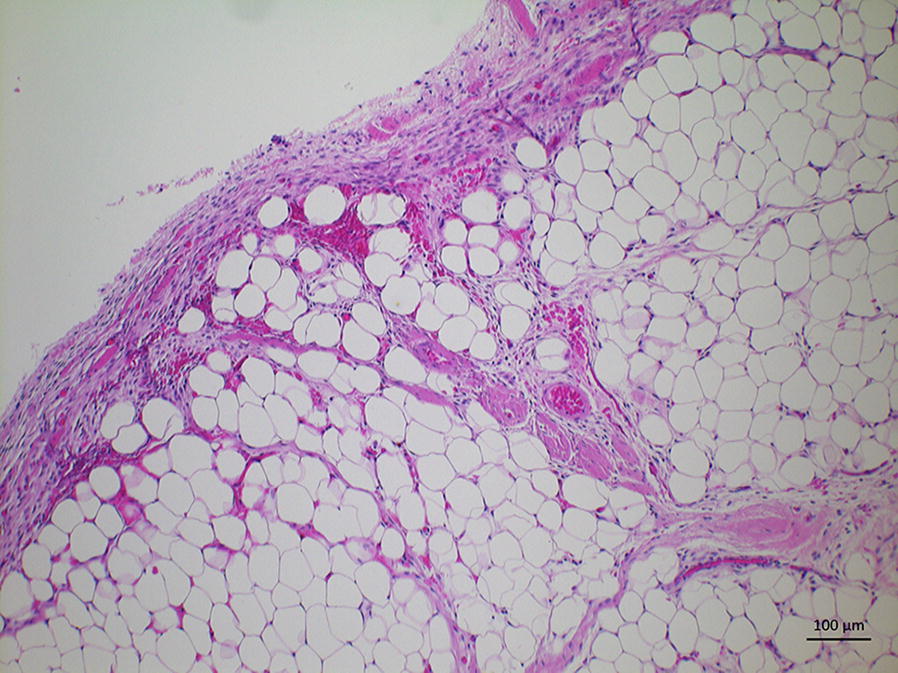
Photomicrograph of the intratracheal mass. The mass consisted of mature uniform lipocytes and no signs of malignancy were detected. All excision margins were clean. The mass was surrounded by a distinct relatively thick capsule

The dog was placed in dorsal recumbency and neck in extended position. Before the incision, the surgical area was infiltrated with lidocaine (Lidocain 20 mg/mL; Orion Oyj Orion Pharma). The cervical trachea was approached via ventral midline approach. The centre of the incision was approximately 10 cm from the larynx. The dissection was continued along the left side of the trachea as the endoscopic examination suggested that the mass extended more to the left side of the dorsal membrane. Oesophagus, nerves and vessels were avoided during the dissection. A stay suture was placed around the tracheal cartilage to facilitate rotation of the trachea to the right. The tracheal mass was localised using palpation and a longitudinal incision was made to the dorsal membrane next to the tumour margin. On the right side, the mass was attached to the dorsal membrane and on the left side to the site where the dorsal membrane joins cartilage. The tumour was resected from the dorsal membrane with narrow margins of healthy looking tissue. The right-sided dorsal membrane was sutured to the cartilage edges and annular ligaments on the left side using simple interrupted 3-0 polydioxanone (PDS, Ethicon) appositional sutures placed 2–3 mm from the wound edge. The last sutures were preplaced before tightening. Closure of the wound was performed in a routine fashion. On macroscopic evaluation the mass resembled fat tissue. The mass was formalin fixed and processed for histology at which it was identified as a lipoma (Fig. 3).

The dog was hospitalised for 24 h after surgery for postoperative observation. Respiratory signs were relieved immediately after surgical removal of the tumour and postoperative recovery was uneventful.

**Fig. 4:**
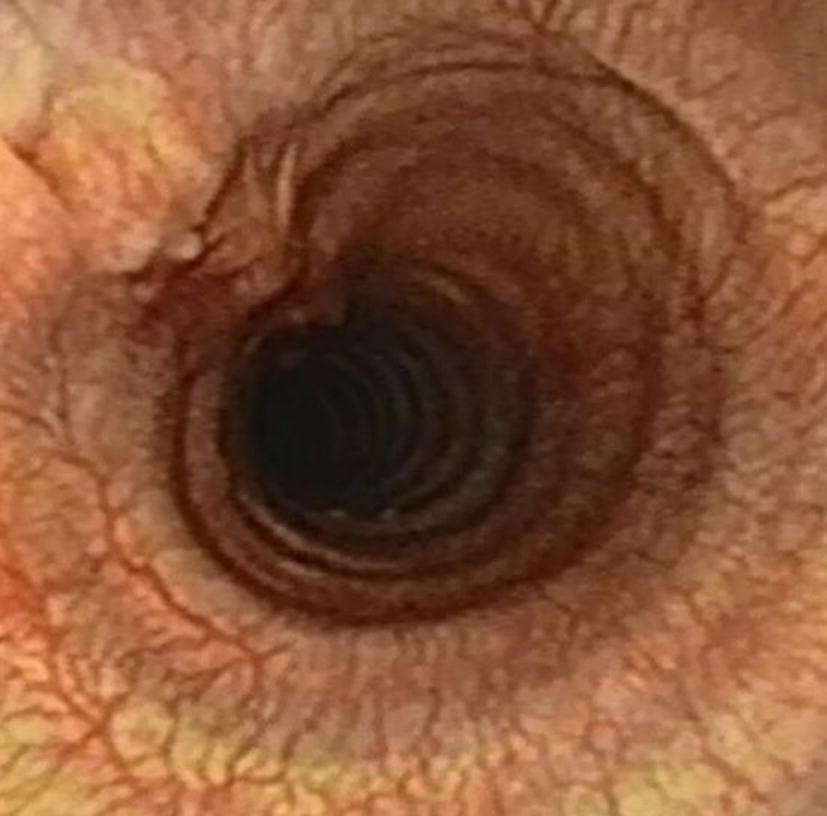
Endoscopic view of the surgical area 6 weeks after surgery

At the control visit 6 weeks after the operation, the dog was free of symptoms and there were no abnormal findings on physical examination. In the control endoscopic examination, the surgical area had healed well with minimal scar tissue formation and no tracheal stenosis (Fig. 4). The owner was contacted by telephone 9 months after the surgery and reported the dog to be asymptomatic.

## Discussion and conclusions

To our knowledge this is the first reported case of canine tracheal lipoma. The finding is exceptional as the majority of tracheal tumours reported thus far in both veterinary and human literature have been malignant. Lipomas are among the most common tumours of the skin and soft tissue in dogs; lipomas in other parts of the canine respiratory tract such as the lungs, larynx and epiglottis have been described in case reports [20–23].

Preoperative cytological or histopathological diagnosis of tumour type is essential for the prognosis and treatment of the patient; it affects the treatment modality chosen as well as determines surgical margins necessary for successful removal [24]. In our patient, we chose endoscopic FNA as diagnostic approach instead of forceps biopsy or the combination of both, because a severe tracheal obstruction was detected and there was a risk that intratracheal bleeding from biopsy site would exacerbate clinical signs. However, cytology did not provide accurate diagnosis of tumour type, possibly because the capsule surrounding the lipoma was not penetrated during sampling. Similarly, Arslan et al. [30] has reported that FNA was diagnostic in only four of 11 people with parotid lipoma. The major advantage of forceps biopsies is that the specimens are suitable for histological examination. However, biopsies may be too superficial, especially in cases of submucosal or encapsulated lesions [25]. Endoscopic FNA is capable of reaching the deeper layers of the lesion, but may not be representative of the tumour, especially if inadequate numbers of cells are collected [25]. In humans, similar diagnostic sensitivities (68–91%) have been reported for both forceps biopsies and FNA of central airway lesions [25]. As recommended in humans, the combination of these techniques would have been ideal also in our patient [25].

Staging and evaluation of tumour invasion provides important prognostic information and aids surgical planning. In the described case, pulmonary nodules or enlarged lymph nodes were not detected in thoracic radiographs. However, it has been shown that CT is more sensitive in detecting pulmonary metastasis than radiography and in our case a CT examination would have provided valuable information on possible lymph node or lung metastasis, enabled sampling of these lesions and would have displayed the extent of the tumour outside endoscopically visible trachea [26–28]. Additionally, in this case CT examination could have raised a suspicion of lipoma by demonstrating tissue density typical for fat [29, 30]. CT was not performed in our patient because of financial constraints.

In previous reports, canine tracheal tumours have been excised by endoscopic snaring of the tumour, blunt dissection or tracheal resection and anastomosis [1, 24]. In humans, airway lipomas have been successfully treated by endoscopic resection or laser therapy [17]. In our case, surgical planning was challenging because the tumour type was not fully known as the FNA cytology obtained was insufficient to rule out malignancy. Considering that tracheal tumours are most often malignant, a tracheal resection and anastomosis would have been surgical approach of choice for a complete removal of the tumour [31]. However, tracheal resection carries a higher risk of postsurgical complications, including granulations at the anastomotic site, infection, decrease of mucociliary clearance, anastomotic separation and tracheal stenosis [32, 33]. In our patient, local surgical resection was technically possible because the tumour originated from the dorsal membrane, and this approach was chosen even though it was acknowledged that in case of malignancy, the margins were likely inadequate. Minimally invasive endoscopic techniques such as snaring or laser ablation would have been optimal if the benign nature of the tumour was fully known prior to surgery [34, 35].

This report adds lipoma as a differential diagnosis for an intraluminal tracheal tumour in dogs and shows that surgical excision may be curative.
